# Experimental assessment of out‐of‐field dose components in high energy electron beams used in external beam radiotherapy

**DOI:** 10.1120/jacmp.v16i6.5616

**Published:** 2015-11-08

**Authors:** Mohamad M. Alabdoaburas, Jean‐Pierre Mege, Jean Chavaudra, Jérémi Vũ Bezin, Attila Veres, Florent de Vathaire, Dimitri Lefkopoulos, Ibrahima Diallo

**Affiliations:** ^1^ Inserm (CESP Centre for Research in Epidemiology and Population Health) U1018, Radiation Epidemiology Team Villejuif France; ^2^ Institut Gustave Roussy Villejuif France; ^3^ Université Paris‐Sud Orsay France; ^4^ Equal‐Estro Laboratory Villejuif France

**Keywords:** electron beam therapy, out‐of‐field dose, electron applicator

## Abstract

The purpose of this work was to experimentally investigate the out‐of‐field dose in a water phantom, with several high energy electron beams used in external beam radiotherapy (RT). The study was carried out for 6, 9, 12, and 18 MeV electron beams, on three different linear accelerators, each equipped with a specific applicator. Measurements were performed in a water phantom, at different depths, for different applicator sizes, and off‐axis distances up to 70 cm from beam central axis (CAX). Thermoluminescent powder dosimeters (TLD‐700) were used. For given cases, TLD measurements were compared to EBT3 films and parallel‐plane ionization chamber measurements. Also, out‐of‐field doses at 10 cm depth, with and without applicator, were evaluated. With the Siemens applicators, a peak dose appears at about 12–15 cm out of the field edge, at 1 cm depth, for all field sizes and energies. For the Siemens Primus, with a $10\times 10{\text{cm}}^{2}$ applicator, this peak reaches 2.3%, 1%, 0.9% and 1.3% of the maximum central axis dose (Dmax) for 6, 9, 12 and 18 MeV electron beams, respectively. For the Siemens Oncor, with a $10\times 10{\text{cm}}^{2}$ applicator, this peak dose reaches 0.8%, 1%, 1.4%, and 1.6% of Dmax for 6, 9, 12, and 14 MeV, respectively, and these values increase with applicator size. For the Varian 2300C/D, the doses at 12.5 cm out of the field edge are 0.3%, 0.6%, 0.5%, and 1.1% of Dmax for 6, 9, 12, and 18 MeV, respectively, and increase with applicator size. No peak dose is evidenced for the Varian applicator for these energies. In summary, the out‐of‐field dose from electron beams increases with the beam energy and the applicator size, and decreases with the distance from the beam central axis and the depth in water. It also considerably depends on the applicator types. Our results can be of interest for the dose estimations delivered in healthy tissues outside the treatment field for the RT patient, as well as in studies exploring RT long‐term effects.

PACS number(s): 87.53.Bn, 87.56.bd, 87.56.J‐

## I. INTRODUCTION

The large improvements in radiotherapy (RT) procedures have led to high survival rates of the patients, so the possible late side effects of the dose delivered to the normal tissues^(1)^ will be a growing concern. More understanding of side effects of RT will require not only improved control of the high doses delivered to the target volumes, but also better knowledge of the unintended but unavoidable lower doses delivered out of the target. In addition, an accurate evaluation of out‐of‐field dose from photon beam or electron beam may be important for RT patients having cardiac implantable devices.^(2)^ To date, most studies on out‐of‐field dose estimation focus on photon beams.^3^, ^4^ Nevertheless, electron beams are still an important component of RT, for treating superficial tumors (at depths <5 cm).^(5)^ In high‐energy electron beam RT (4–25 MeV); applicators with cutouts are used to confine the beams to the target area. Various electron applicators are currently used in clinical routines. The design and materials of these applicators differ by manufacturer. The applicators are designed to allow scattered electrons and transmission radiations to escape outside the treatment beam as little as possible. However, it is well known that a non‐ignorable fraction of scattered and transmission radiations can escape from the applicator and induce unintended doses outside the treatment field.^6^, ^7^, ^8^, ^9^, ^10^, ^11^, ^12^

The two main components of out‐of‐field doses outside the applicator are bremsstrahlung photon contamination and scattered electrons.^11^, ^13^ The bremsstrahlung photons can be produced in different structures of the accelerator head and in the patient irradiated volume.^(14)^ The scattered electrons can travel through the applicator parts, or emanate from the scattering foils in air, without interacting with the different parts of the applicator.^(11)^

Several experimental investigations have studied the dose and the leakage levels outside the applicator for older electron applicators.^6^, ^7^, ^8^, ^9^, ^10^ Another study has been performed on the peripheral dose for a modern Varian‐type applicator.^(11)^ Those authors found, for 4 MeV electron beams, a peak dose appearing at about 7 cm from the beam edge outward. Then they focused their study on the dependence of this peak dose on different parameters (such as applicator size, depth, etc.). However, their study was limited to off‐axis distances up to 14 cm from CAX and for depths in water up to 1 cm from phantom surface. Another detailed experimental study has been performed on an applicator type EA200 on the Siemens Primus.^(12)^ A recent investigation, comparing experimental data and Monte Carlo simulations, has been reported for a Varian‐type applicator.^(13)^ In spite of a fair number of studies published on the out‐of‐field dose from high‐energy electron beams, the out‐of‐field doses from electron beams from the Digital Electron Variable Applicator (DEVA) on the Siemens Primus have not yet been studied. In addition, more detailed data are still required about the out‐of‐field doses for the Varian‐type applicator and Siemens‐type applicators Series EA3. So the purpose of this work was to investigate the dose outside the applicator in a water phantom for the three different electron applicator types mentioned above. We have evaluated the dose dependence on applicator size, electron beam energy, depth in water, and off‐axis distance.

## II. MATERIALS AND METHODS

### A. Linear accelerators and associated applicators

**Figure 1:**
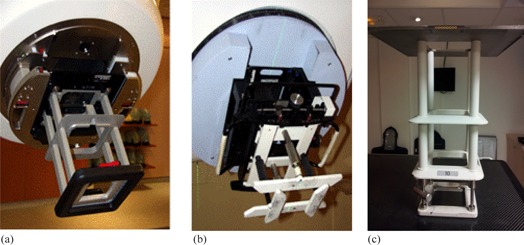
The three different types of applicator used: (a) the Varian‐type, (b) the Siemens DEVA, and (c) the Siemens series EA3.

The measurements were performed on a Varian Clinac 2300C/D (Varian Medical Systems, Palo Alto, CA), a Siemens Primus KD2 (Siemens Healthcare, Malvern, PA), and a Siemens Oncor. The electron applicators of these accelerators have a diaphragm‐type design, differing as shown in Fig. 1. The characteristics of the applicator scrapers (diaphragms) to collimate the electron beam are detailed here for each applicator.

- For the Varian type applicators, Fig. 1(a), three scraper levels provide a range of discrete field sizes from 6 cm×6 cm to 25 cm×25 cm. All scrapers have a 5 cm width and a thickness of about of 2 cm (inside edge) decreasing to about 0.2 cm (outside edge). The material used for all applicators consists of 8.4% aluminum, 1% copper, and 0.02% manganese‐zinc alloy, the rest being zinc.
- For the Digital Electron Variable Applicator (DEVA) on the Siemens Primus, independent collimation in X and Y directions can be set, with field sizes ranging from 4 cm×4 cm to 25 cm×25 cm, Fig. 1(b). The top scrapers have a 5.1 cm width and a 1.2 cm thickness, while the bottom scrapers have a 4.5 cm width and a 1.4 cm thickness. The scrapers are made of brass.
- For the Siemens type applicators Series EA3, Fig. 1(c), adapted on the Oncor, four scraper levels provide a range of discrete field sizes from about 5 cm (diameter) to 25 cm×25 cm. All scrapers have a 5 cm width. The second from the top and the bottom scrapers have a 1.27 cm thickness, while the third from the top has a 1.57 cm thickness. The applicator is made of a brass alloy. For all applicators, the plate at the top level is made of an aluminum alloy and is 0.66 cm thick.

For a given applicator size and beam energy, the automatic setting of the collimation jaws was specifically set for each linac. The values are given in Table 1, where the position of the jaws is tabulated as a function of the electron applicator field size and the nominal electron energy. For the Primus, the jaw positions are fixed per applicator for all energies and cannot be changed by the user. For the Varian and the Oncor, the jaw position changes with both the energy and the applicator field size.

**Table 1:** Settings of the collimator jaw position according to the applicator and the nominal electron beam energy. All field sizes are defined at SSD=100 cm. $R_{100},R_{50}$, and $R_{P}$ values are specific for each electron beam energy

				*Applicator Field Size*		
				6×6 ^a^	($cm^{2}$) 10×10	20×20
*Energy (MeV)*	*R_100_ (cm)*	*R_50_ (cm)*	*R_P_ (cm)*	*Collimator Jaw Settings (cm^2^)*		
*Siemens Primus KD2*						
6	1.40	2.45	3.05	16×16	19×19	27×27
9	2.10	3.55	4.36	16×16	19×19	27×27
12	2.90	4.79	5.83	16×16	19×19	27×27
18	3.50	7.57	9.12	16×16	19×19	27×27
*Varian 2300C/D*						
6	1.34	2.37	3.00	20×20	20×20	25×25
9	2.15	3.60	4.39	20×20	20×20	25×25
12	2.88	5.02	6.01	11×11	14×14	25×25
18	3.45	7.64	9.10	11×11	14×14	22×22
*Siemens Oncor*						
6	1.40	2.47	3.16	13×13	19×19	30×30
9	2.10	3.60	4.50	13×13	19×19	29×29
12	2.80	4.82	5.80	13×13	19×19	28×28
14	3.10	5.21	6.30	13×13	19×19	27×27

a For Siemens Oncor, there is no 6 cm×6 cm applicator but a circular applicator with 5 cm diameter.

### B. Dosimeters

Powder‐type thermoluminescent dosimeters (TLD‐700; Harshaw Chemical Company, Solon, OH) were used. The powder was encapsulated into opaque polyethylene cylindrical capsules (IAEA type) having 1 mm thick walls, about 3 mm inner diameter and about 20 mm inner length. Each dosimeter contained about 180 mg of powder, allowing five readings per point of measurement. The dose‐induced signal from the dosimeters was read out using a PCL‐3 (Fimel, Vélizy, France) automated TLD reader. All TLDs were prepared and read by Equal‐Estro Laboratory^(15)^ (Equal‐Estro, Villejuif, France). The choice of TLD‐700 material allows avoiding secondary neutrons' contributing to the measurements at the highest energies.

The dose calibration of TLD signals in electron beams was performed using a parallel‐plate ionization chamber (NACP‐02, DFA0005809, Scanditronix, IBA Dosimetry GmbH, Schuarzenbruck, Germany). A calibration coefficient was applied to the TLD's signal for each energy. This coefficient was defined in reference conditions: at the field center, for a 10 cm×10 cm applicator and a delivered dose of 2 Gy, for each electron beam at the corresponding maximum depth dose.^(16)^ No correction was applied to the TLD signal for a possible change in response arising from the spectral change between in‐field and out‐of‐field measurement conditions.

The TLD measurements were compared to the Gafchromic EBT3 film measurements and plane‐parallel ionization chamber NACP measurements.

### C. Measurement of out‐of‐field dose in a water phantom

**Figure 2:**
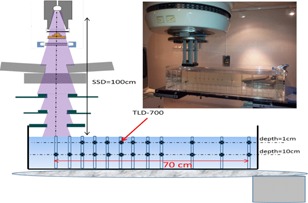
Experimental setup for the measurements with TLD700.

To assess the doses outside the electron applicators, measurements were made at depths of 1 cm and 10 cm, in a 110 cm×40 cm×25 cm water tank, Fig. 2. The measurements were performed from 5 cm beyond the optic field edge to 70 cm from the beam central axis (CAX) for measurements at 1 cm depth, whereas the measurements at 10 cm depth were performed from the beam central axis (CAX) to 70 cm. All measurements were made along the y‐plane direction. The source‐to‐surface distance (SSD) was set to 100 cm for all measurements. The TLDs were irradiated with a sufficient MU number to obtain a lowest out‐of‐field signal more than five times the background signal (without irradiation). The measurements were normalized to the central axis maximum dose (Dmax).

To assess the applicator size and beam energy dependences, the measurements were made for three applicator sizes: 6 cm×6 cm (5 cm diameter for the Oncor), 10 cm×10 cm, and 20 cm×20 cm, for beam energies 6 MeV, 9 MeV, 12 MeV, and 18 MeV (14 MeV for the Siemens Oncor).

### D. Depth dependence

**Figure 3:**
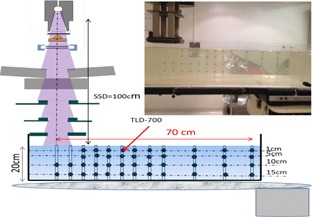
Experimental setup for the depth measurements with TLD 700.

Depth‐dose measurements were performed with a 10 cm×10 cm applicator, at SSD=100 cm, on the Siemens Oncor operated at 14 MeV, as well as on the Varian operated at 9 MeV and 18 MeV. For the Varian, the measurement depths were 1 cm, 5 cm, 10 cm, and 15 cm in the water tank as a function of the off‐axis distances ranging from the beam central axis up to 70 cm, along the y‐plane direction, as shown in Fig. 3. For the Siemens Oncor, the measurements as a function of the depth were achieved at chosen off‐axis distances 17.5 cm, 25 cm, and 35 cm from CAX. All the measurements were normalized to Dmax.

### E. Assessment of applicator contribution to out‐of‐field bremsstrahlung dose

To estimate the applicator contribution to out‐of‐field bremsstrahlung dose, TLD700 measurements were performed on the Varian operated at 12 MeV and 18 MeV, at 10 cm depth in the water tank, where only a bremsstrahlung component would be expected to contribute to the dose. These measurements were made without applicator, and the jaw collimators were kept at the setting corresponding to a 10 cm×10 cm applicator, as mentioned in Table 1, for off‐axis distances from beam central axis (CAX) to 70 cm, at SSD=100 cm. To obtain the applicator contribution, subtraction was made between with and without applicator measurements.

### F. Comparison of TLD measurements to film and ionization chamber (IC) measurements

**Figure 4:**
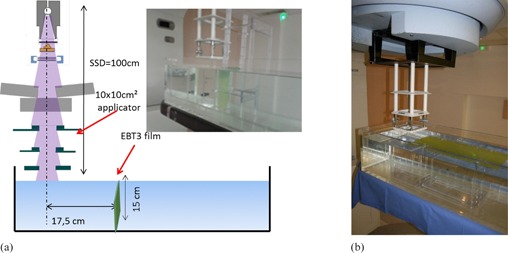
Experimental setup for the EBT3 film measurements (a) as a function of depth, (b) as a function of off‐axis distance.

In order to compare the TLD measurements with film and plane‐parallel ionization chamber measurements, measurements using EBT3 films were performed on the Siemens Oncor, with the 10 cm×10 cm applicator, at SSD=100 cm, in the same water tank. In the one case, EBT3 films were vertically positioned and the measurements were performed at 17.5 cm from CAX as a function of the depths ranging from 0 cm to 15 cm, as shown in Fig. 4(a), for 14 MeV. In the other case, the EBT3 films were horizontally positioned and the measurements were performed at 1 cm depth as a function of off‐axis distances ranging from 10 cm to about 55 cm from CAX, as shown in Fig.4(b), for 12 MeV (the energies were chosen arbitrarily in these measurements). The film was fixed by a Plexiglas support, specifically designed for this purpose. For these measurements, the MU number was chosen to obtain a lowest out‐of‐field dose more than 15 cGy. The measurements were normalized to the central axis maximum dose (Dmax). The dose response of the EBT3 films was established according to the 3‐channel protocol. As we used these films and software for pretreatment verifications, the response of a specific batch (A03031407) was established in a 6 MV standard beam (Clinac 21EX, Varian). The reference absorbed dose was determined according to the IAEA TRS 398 protocol. The above measurements were also performed using a plane‐parallel ionization chamber NACP. The out‐of‐field ionization readings were normalized to the central axis maximum ionization.

## III. RESULTS

### A. Measurements of out‐of‐field dose in the phantom plane

**Figure 5:**
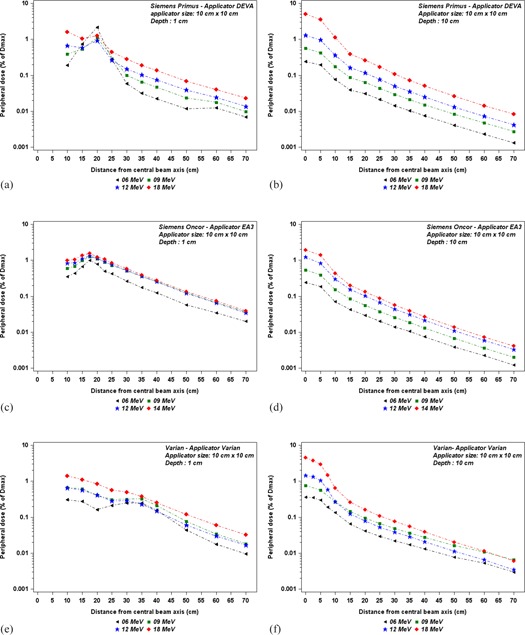
Out‐of‐field dose variations, according to distance from the beam central axis, for different energies, 10 cm×10 cm applicator. The measurements were performed for ((a) and (b)) the Siemens Primus at depths of 1 cm and 10 cm, for ((c) and (d)) the Siemens Oncor at depths of 1 cm and 10 cm, and for ((e) and (f)) the Varian at depths of 1 cm and 10 cm.

Figure 5 illustrates our results on out‐of‐field dose variations according to the distance from beam central axis for 10 cm×10 cm applicators, at 1 cm and 10 cm depths. For the Siemens Primus, we observed, as off‐axis distance increases the out‐of‐field dose increases up to a maximum at about 20 cm from the beam central axis (see Fig. 5(a)). This peak is more pronounced for the lowest energy and reaches 2.3%, 1.1%, 0.9%, and 1.3% of Dmax for 6, 9, 12, and 18 MeV electron beams, respectively. Beyond the peak, out‐of‐field dose decreases as a function of the lateral distance; the decrease being more pronounced for the lowest beam energies.

For the same applicator size, with the Siemens Oncor, as shown in Fig. 5(c), the out‐of‐field dose increases as off‐axis distance increases to form a peak dose at about 17.5 cm from the beam central axis, which reached 0.8%, 1%, 1.4%, and 1.6% of Dmax for 6, 9, 12, and 14 MeV, respectively. Then, the out‐of‐field dose decreases exponentially with distance.

For the Varian Linac, the out‐of‐field dose decreases continuously with increasing distance. These doses, at 17.5 cm from the beam central axis, are 0.3%, 0.6%, 0.5%, and 1.1% of Dmax for 6, 9, 12, and 18 MeV, respectively. No peak dose is evidenced for the Varian applicator, except for the 6MeV energy beam; where one can see a small peak at 30 cm from the beam central axis, as shown in Fig. 5(e).

Figures 5(b), 5(d), and 5(f) depict the off‐axis dose profiles at 10 cm depth, for 10 cm×10 cm applicators. For the same beam energy, no significant difference is found between the three applicators. From the CAX, the dose profiles first slowly decrease as a function of off‐axis distance up to the field edge; afterward, they rapidly decrease to form the field penumbra, and then, outside the applicator, decrease exponentially as a function of off‐axis distance. For the Siemens Primus, as shown in Fig. 5(b), the out‐of‐field dose at 15 cm from the beam central axis is about 0.04%, 0.09%, 0.18%, and 0.35% of Dmax for 6, 9, 12, and 18 MeV, respectively. For the Oncor, as shown in the Fig 5(d), the out‐of‐field doses at 15 cm from beam central axis are 0.04%, 0.09%, 0.15%, and 0.2% of Dmax for 6, 9, 12, and 14 MeV, respectively. For the Varian, at the same position, the out‐of‐field dose reaches 0.07%, 0.14%, 0.12%, and 0.33% of Dmax for 6, 9, 12, and 18MeV, respectively, as shown in Fig. 5(f).

**Figure 6:**
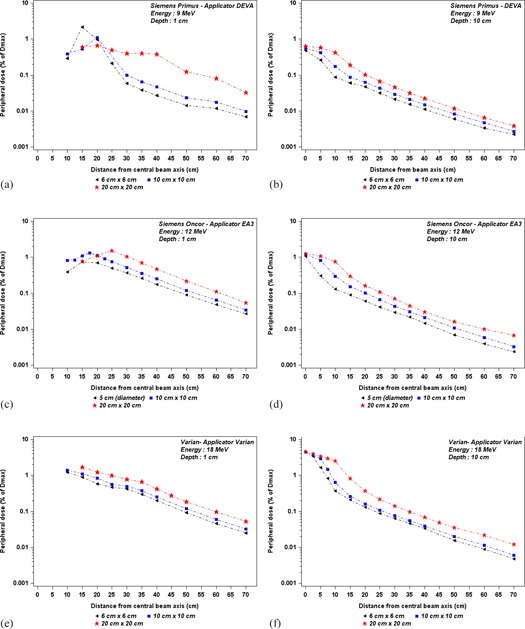
Out‐of‐field dose variations, according to distance from the beam central axis, for different applicator sizes at given energy for each accelerator. The measurements were performed ((a) and (b)) for 9 MeV Siemens Primus at depths of 1 cm and 10 cm, ((c) and (d)) for 12 MeV Siemens Oncor at depths of 1 cm and 10 cm, and ((e) and (f)) for 18 MeV Varian at depths of 1 cm and 10 cm.

Figure 6 illustrates the out‐of‐field dose dependence on applicator size, at 1 cm and 10 cm depths in water, as a function of off‐axis distance, for a given energy. As shown in Fig. 6(a), for the Siemens Primus, the dose peak value of the out‐of‐field dose increases when the applicator size decreases. Beyond the peak, the out‐of‐field dose increases with applicator size. For the Oncor and the Varian, the out‐of‐field dose increases with applicator size, as shown in Fig. 6(c), Fig. 6(d), and Fig. 6(e). Figures 6(b), 6(d), and 6(f) show out‐of‐field dose dependence on applicator size, at 10 cm depth as a function of off‐axis distance, for a given energy. The out‐of‐field dose increases with applicator size for all out‐of‐field distances. However, the increase of out‐of‐field doses with the applicator size becomes less significant when the origin of the off‐axis distances is taken from the optic field edge.

### B. Depth dependence

**Figure 7:**
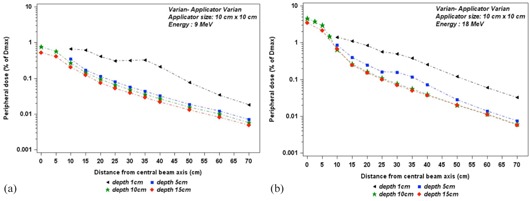
Out‐of‐field dose variations, according to distance from the field center, at different depths in a water tank, for applicator size of 10 cm×10 cm. The measurements were performed (a) for 9 MeV and (b) for 18 MeV electron beams on the Varian.

**Figure 8:**
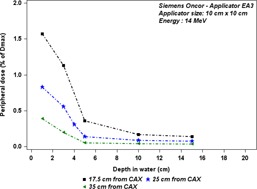
Out‐of‐field dose penetration, according to depth in a water tank, for applicator size 10 cm×10 cm. The measurements were performed with TLD700, at off‐axis distances of 17.5 cm, 25 cm, and 35 cm from field center, for the Oncor linac operated at 14 MeV.

Figures 7(a) and 7(b) show the out‐of‐field dose variations as a function of off‐axis distances for different depths in water tank for two energies, 9 MeV and 18 MeV. We noticed the dose profiles for 9 MeV measured at 5 cm, 10 cm, and 15 cm decrease similarly with off‐axis distance, whereas the dose profile measured at 1 cm depth decreases more slowly with off‐axis distance. Figure 8 shows the out‐of‐field dose variations as a function of depths in a water tank at off‐axis distances 17.5 cm, 25 cm, and 35 cm from CAX, for Siemens Oncor operated at 14 MeV. We notice the dose rapidly decreases with depth until about 5 cm depth, and then starts to decrease slowly.

### C. Assessment of applicator contribution to out‐of‐field bremsstrahlung dose

**Figure 9:**
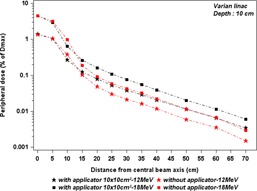
Out‐of‐field dose variations, according to distance from the field center, at depth of 10 cm in a water tank, for 14 cm×14 cm jaws aperture, with and without electron applicator. The measurements were performed on the Varian linac.

Figure 9 shows the contribution of the bremsstrahlung dose produced in the electron applicator to the total bremsstrahlung dose component in‐field and out‐of‐field. We noticed that inside the applicator this contribution does not exceed 7% of total bremsstrahlung dose component, whereas outside the applicator it represents about 55% of total bremsstrahlung dose for tow energies 12 MeV and 18 MeV. In contrast, at field edge, the total bremsstrahlung dose without applicator is higher than with applicator.

### D. Comparison of TLD measurements with film and ionization chamber (IC) measurements

**Figure 10:**
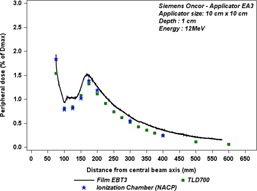
Comparison between out‐of‐field doses measured with three different dosimeters, at 1 cm depth as a function of off‐axis distance for the Siemens Oncor linac operated at 12 MeV.

Figure 10 shows a comparison of TLD measurements with film and ionization chamber (IC) measurements, at 1 cm depth, as a function of off‐axis distance. We notice a good agreement between TLD700 and the ionization chamber measurements, with an average difference within ± 5%. In contrast, the EBT3 film measurements overestimated the dose in comparison with TLD700 and ionization chamber measurements, by about 0.1% of Dmax for all points, as reported in Table 2.

**Figure 11:**
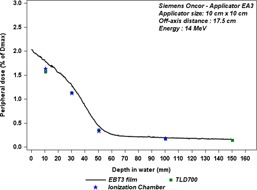
Comparison between the out‐of‐field doses measured with three different dosimeters, at 17.5 cm from CAX as a function of depth for the Siemens Oncor linac operated at 14 MeV.

The comparison of TLD measurements with ionization chamber (IC) and film measurements as a function of the depth is shown in Fig. 11. The measurement discrepancies between IC and TLD700 reach up to 9% for 5 cm depths. The measurement discrepancies between IC and the film is about 8%–13% for shallow depths, and it reaches up to 26% at 5 cm depth, as reported in Table 3.

**Table 2:** Out‐of‐field dose values in percentage of Dmax, at different distances from CAX, measured with TLD700, EBT3 films, and an ionization chamber (IC) (NACP), at 1 cm depth, for the Siemens Oncor linac operated at 12 MeV. Discrepancies between IC, TLD, and film measurements are given as mean relative differences

*Distance from Central Axis (cm)*	*Out‐of‐field Dose (%Dmax)*			*Relative Difference TLD & IC*	*Relative Difference Film & IC*
	*TLD*	*Film*	*IC*		
10	0.82	0.93	0.78	5%	17%
17.5	1.32	1.49	1.38	−3%	7%
30	0.52	0.64	0.54	−4%	17%
40	0.25	0.35	0.24	4%	37%

**Table 3:** Out‐of‐field dose values in percentage of Dmax for different depths, measured with TLD700, EBT3 films, and an ionization chamber (IC) (NACP), at 17.5 cm from CAX, for the Siemens Oncor linac operated at 14 MeV. Discrepancies between IC, TLD, and film measurements are given as mean relative differences

*Depth (cm)*	*Out‐of‐field Dose (%Dmax)*			*Relative Difference TLD & IC*	*Relative Difference Film & IC*
	*TLD*	*Film*	*IC*		
1	1.57	1.77	1.63	−4%	8%
3	1.13	1.28	1.12	1%	13%
5	0.36	0.43	0.33	9%	26%
10	0.17	0.19	0.18	−6%	6%

## IV. DISCUSSION

The out‐of‐field doses in high‐energy electron beams are evaluated for three different electron applicator types used in daily practice. We have investigated the dependence of this dose on different parameters, such as the applicator size, the electron beam energy, the depth, and the off‐axis distance. The applicator contribution to out‐of‐field bremsstrahlung dose has also been experimentally estimated.

The doses measured outside the applicator at 1 cm depth in the water phantom exhibit a local peak dose for both Siemens machines. This peak does not appear for the measurements at 10 cm. Hence, we suppose that this peak is essentially due to the electrons.

For the Siemens Primus, as this peak is more pronounced when the energy and applicator size decrease, we expect this peak is due to the electrons escaping directly through the applicator gaps, rather than being due to the electrons scattered through the collimator edges, as, in that case, the lateral spread of electrons emanating from scattering foils would increase when beam energy decreases.

For the Siemens Oncor, the peak dose increases with both beam energy and applicator size. Regarding the upper scraper in Siemens applicator EA3, as it is made of aluminum alloy with a thickness of 0.66 cm, some of the primary electrons can transmit through it. That can explain the increase of the peak dose with beam energy. The region between the field edge and the peak is relatively well shielded by the other scrapers, which can decrease the dose caused by escaping electrons. The shape of the depth dose profiles measured with the film at 17.5 cm from the CAX shows that the electrons component is much bigger than the photon component for shallow depths (Fig. 11 and Fig. 8). These electrons have a high penetration range, which can reach about 5 cm depth, and this can be compared to the practical range inside the field for the 14 MeV beam, as reported in Table 1. Afterward, the dose decreases very slowly, which represents bremsstrahlung yield. As the penetration range dependence on the off‐axis distance is marginal outside the applicator (Fig. 8), as one can expect, the variation of electron energy as a function of the lateral distances is also small. But the decrease of dose with depth becomes slower for further off‐axis distances from the CAX, which could be due to the increase of the obliquity angle of the incident electrons with off‐axis distances.

For the Varian, no peak dose was observed (for energies more than 6 MeV) in agreement with Chow and Grigorov.^(11)^ The depth dose for Varian, presented in Fig. 7(b), shows that some of electrons can penetrate to 5 cm depth in the water; hence the penetration depth of electrons outside the applicator can be about half of that inside the applicator (R_p_ for 18 MeV). Therefore, one can expect that the electrons scattered outside the field emanate from the collimator edges, rather than escaping directly from scattering foils, as Lax and Brahme^(17)^ have reported that the scattered electrons from a collimator edge for an electron beam could have an average energy of about 40% of the incident electron energy.

Previous experimental investigations have reported that, at about 10 cm outward from the beam edge, the out‐of‐field dose may reach 5% of maximal central axis dose^(12)^ for the applicator type EA200 on Siemens Primus, this large discrepancy between those findings and our results obtained on the same linac (Siemens Primus) being due to the applicator type used. In our study, the DEVA applicator is used. Iktueren et al.^(18)^ have found that the out‐of‐field dose on the Oncor linac can be between 1.7% and 4.1% at 1 cm depth, increasing with beam energy and applicator size. However, the authors have not specified the applicator type used in that study, making any comparison between their measurements and our results difficult. Chow and Grigorov assessed the out‐of‐field dose outside Varian‐type applicators set on a Varian 21 EX linac. They found that the dose peak, at 1 cm depth, for the 4 MeV beam, was about 12 cm from the CAX for field size 10 cm×10 cm, and was 1.5% of the central axis maximal dose.

A recent investigation with Monte Carlo has been reported by Shimozato et al.^(13)^ They calculated the fluence distributions and the dose distributions outside the applicator for a Varian‐type applicator, Clinac 2100CD. They found that the out‐of‐field dose was about 1% of maximal central axis dose for 16 MeV electron beam. Our findings on Varian linac are in agreement with their data.

The out‐of‐field doses at 10 cm depth for electron beams are significant and may be comparable, in certain circumstances, to values reported for photon beams, about 55% of this dose originating from the electron applicator. The dose profiles, at 10 cm depth, are similar in all machine types for a given energy and applicator size. The doses decrease exponentially with off‐axis distance and increase with energy, which can be understood from the characteristics of bremsstrahlung produced by impinging an electron beam on a target. Our measurements for in‐field bremsstrahlung doses at 10 cm depth on the Varian 2300 C/D are in agreement with those reported by Zhu et al.^(14)^

In Fig. 5(f) we can observe that the doses outside the applicator at 10 cm depth for energy 9 MeV are higher than for 12 MeV for the 10 cm×10 cm applicator, contrary to the in‐field doses. This can be explained by the irradiated volume quantity in the applicator material. This irradiated volume, for the same applicator size, increases when the jaw aperture increases. As shown in Table 1, for a 10 cm×10 cm applicator, the jaw apertures are 14 cm×14 cm and 20 cm×20 cm, for 12 MeV and 9 MeV, respectively. As the out‐of‐field dose at 10 cm depth is essentially issued from the electron applicator, which represents about 55% of the total out‐of‐field bremsstrahlung dose (Fig. 9), this influence is more significant outside the applicator than inside the applicator, where, in the latter, the bremsstrahlung dose is essentially issued from scattering foils.

The uncertainty estimations of these measurements are complex and remain a challenge, because of the lack of information about the radiation spectrum and low dose delivered in this area. However, Shimozato et al.^(13)^ have reported that the bremsstrahlung spectrum outside Varian applicators have a peak around 250 KeV, 350 KeV, 500 KeV, 750 KeV, and 1 MeV for 4 MeV, 6 MeV, 9 MeV, 12 MeV, and 16 MeV electron beams, respectively. So we used three different dosimeter types to compare their responses. The EBT3 dosimeter overestimates the dose by 7% as compared to the other dosimeters at peak dose. This overestimation increases with off‐axis distance to reach 37% (for the lowest doses), but it remains small in absolute terms. In Fig. 11, the measurements with EBT3 film as a function of depth show that film also overestimates the dose by about 8% as compared to IC measurement at 1 cm depth, this overestimation increases with the depth up to 26% at 5 cm depth, where very low‐energy electrons will be present. The discrepancy between IC measurement and two other dosimeter measurements is about ±6% at 10 cm depth, where only bremsstrahlung photons are expected to be present.

Mobit et al.^(19)^ have reported that the uncertainty about absolute dose in electron beam achieved by a TLD100 (rod and chip) can reach up to 10% and more, when one combines all the differences issuing from energy correction factors, TLD thicknesses, and irradiation depth.

Su et al.^(20)^ have found that the electron energy dependence of EBT films was within ±4% for 6−20 MeV electron beams. However, energy dependence and dose dependence of EBT films become more significant when both beam energy and dose quantity are lower.^21^, ^22^ This can explain the increasing discrepancies between the film and the two other dosimeters with off‐axis distances, as well as with depth.

## V. CONCLUSIONS

This work experimentally analyzes out‐of‐field doses from high‐energy electron beams used in RT, for three different applicator types. It shows that, depending on beam energy, applicator size and type, off‐axis distance, and depth in water, in general, out‐of‐field doses from the electron beam increase with beam energy and applicator size and decrease with off‐axis distance and depth. For Siemens machines, a peak dose at 1 cm of depth and at about 12−15 cm from field edge was found. Contrary to the Siemens Oncor, for Siemens Primus this peak dose becomes more pronounced when both beam energy and applicator size decrease.

Estimating doses outside the treatment field for electron beams is important due to possible damage to sensitive organs such as the eye and the thyroid if a peak dose occurs.

These data can be of interest because, to date, out‐of‐field doses with electron beams are not fully accounted for by commercial TPS.

## ACKNOWLEDGMENTS

The first author, Mohamad M. Alabdoaburas, has a PhD study scholarship from the Franco‐Syrian Program. This study was supported by INSERM Plan Cancer, PeriDoseQuality project (number C14017LS), and by the European Commission, FP7‐Health, PanCareSurFup project (grant agreement number 257505).
